# Area under Precision-Recall Curves for Weighted and Unweighted Data

**DOI:** 10.1371/journal.pone.0092209

**Published:** 2014-03-20

**Authors:** Jens Keilwagen, Ivo Grosse, Jan Grau

**Affiliations:** 1 Institute for Biosafety in Plant Biotechnology, Julius Kühn-Institut (JKI) – Federal Research Centre for Cultivated Plants, Quedlinburg, Germany; 2 Institute of Computer Science, Martin Luther University Halle–Wittenberg, Halle (Saale), Germany; 3 German Centre for Integrative Biodiversity Research (iDiv) Halle-Jena-Leipzig, Leipzig, Germany; Indiana University Bloomington, United States of America

## Abstract

Precision-recall curves are highly informative about the performance of binary classifiers, and the area under these curves is a popular scalar performance measure for comparing different classifiers. However, for many applications class labels are not provided with absolute certainty, but with some degree of confidence, often reflected by weights or soft labels assigned to data points. Computing the area under the precision-recall curve requires interpolating between adjacent supporting points, but previous interpolation schemes are not directly applicable to weighted data. Hence, even in cases where weights were available, they had to be neglected for assessing classifiers using precision-recall curves. Here, we propose an interpolation for precision-recall curves that can also be used for weighted data, and we derive conditions for classification scores yielding the maximum and minimum area under the precision-recall curve. We investigate accordances and differences of the proposed interpolation and previous ones, and we demonstrate that taking into account existing weights of test data is important for the comparison of classifiers.

## Introduction

In both theoretical and applied machine learning, assessing the performance of a classifier is of fundamental importance as a crucial step in model selection and comparison [1]. During the last decades, a plethora of performance measures has been proposed and extensively used [2].

Several of these performance measures [3] are scalar values computed from a single confusion matrix. However, as the confusion matrix depends on some arbitrarily chosen classification threshold, comparisons based on such performance measures are often flawed unless at least one cell of the confusion matrix is fixed. In addition, even in such cases where for instance the sensitivity is fixed, the result of the comparison may be different for other thresholds and sensitivities.

Varying the threshold leads to a series of confusion matrices. In case of binary classification, this series of confusion matrices can be visualized by curves, which can then be compared quantitatively by the area under curve (AUC). One popular curve is the receiver operating characteristics (ROC) curve, which plots the true positive rate (sensitivity, recall) against the false positive rate (1 - specificity) [4], [5]. Despite its popularity, the ROC curve has some drawbacks including the decoupling from the class skew [6].

For this reason, the precision-recall (PR) curve [7], which plots the precision (positive predictive value) against the recall (true positive rate) and is equivalent to the false discovery rate curve [8], has gained increasing attention during the last years. Hence, the area under the precision-recall curve (AUC-PR) and performance measures approximating the AUC-PR including R-precision, average precision, and 11-point interpolated average precision also gained increasing attention [9], [10]. These performance measures have been widely used in diverse fields such as computer vision [11], computational biology [12], [13], information retrieval [14], medicine [15], and natural language processing [16], [17].

For computing the AUC-PR and AUC-ROC, the interpolation between two adjacent points of the curve is based on a linear interpolation between the underlying confusion matrices [7]. While for ROC curves this interpolation leads to a linear interpolation of the curve, it is in most cases non-linear for PR curves. The common method for computing PR curves and their AUC-PR for unweighted data is a discrete interpolation along the true positives [7].

In recent years, soft-labeling has gained increased attention, as for many classification problems the labeled input data are associated with some measure of confidence, generically denoted as *weights* in the following. In this paper, we consider as weights values reflecting soft class labels resulting from uncertainty of class labels, some measured signal, or multiplicities of data points, since the methods proposed in this paper are applicable to all these types of weights. We provide a formal definition of the weights considered in section.

While weights are widely used for learning classifiers from training data, the assessment of classifier performance on test data is often restricted to the hard-labeled or unweighted case. However, the determination of a single confusion matrix for a given threshold is straight-forward, where the entries of the confusion matrix are accumulated weights. Hence, it is also straight-forward to compute scalar performance measures as for instance precision and recall for the weighted case. Consequently, the supporting points of ROC and PR curves can also be computed for weighted data, ROC curves and AUC-ROC can be derived as for the unweighted case due to the linear interpolation.

Aiming at computing the AUC-PR for weighted data, it is unclear how to interpolate between such real-valued confusion matrices. For this reason, previous publications considering weighted test data resorted to approximations of the AUC-PR. Examples for such approaches are average precision [18] or an approximation of PR curves for uncertain class labels specific to alternative labelings of image boundaries by several individuals [19]. Here, we propose a continuous interpolation between the points of the PR curve, and we use a piecewise-defined function for computing the AUC-PR by a piecewise explicit integration along the recall values. This approach allows for computing AUC-PR for unweighted as well as weighted data.

In a case study, we investigate differences between the AUC-PR computed using the continuous and the discrete interpolation. Furthermore, we investigate if taking into account given weights when computing AUC-PR may lead to different conclusions when comparing classifiers or when doing model selection based on this performance measure.

## Methods

In this section, we first formally define weights and then revisit confusion matrices. In the remainder of this section, we revisit discrete interpolations for PR curves, propose a generalization yielding a continuous interpolation, and finally show how this interpolation can be applied to weighted data.

### Weights Definition

We consider the case of binary classification and denote the two classes by *foreground* (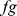) and *background* (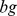). We further consider a sample of data points 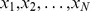 from these two classes. In case of unweighted data, each data point is assigned to exactly one class, either to the foreground class or to the background class.

In this paper, we additionally consider weighted data, where the following types of weights may be assigned to data point 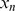:

i) Soft class labels 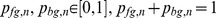 indicating uncertainty in the class labels, where 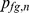 and 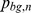 reflect the probability that data point 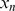 belongs to the foreground class and the background class, respectively [18]–[20].
ii) Values 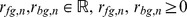, which reflect the strength of some measure of confidence for data point 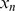 in the two classes. Such measures of confidence are either some experimental measurement or are assigned to the data points by expert knowledge. For instance, the values 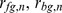 could be the expression values of gene 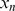 in cancerous (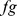) and normal (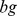) tissue [21].
iii) Multiplicities 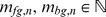 of data point 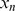, where a data point (or different data points indistinguishable by the features considered) occurs with certain frequencies, including 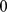, in one or both of the classes. For instance, the values 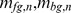 could be the number of occurrences of a short sequence 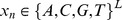 in foreground and background data sets with 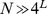 data points [22], [23].

Since the methods presented in the remainder of this paper are applicable to all these types of weights, we generically refer to such soft labels 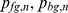, measures of confidence 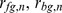, and multiplicities 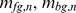 as *weights*, and we denote the weight of data point 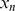 for the foreground class by 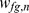 and for the background class by 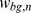. Integrating the above definitions, we derive that each data point has a foreground and background weight 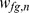 and 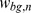, respectively, which are non-negative but may be equal to zero if data point 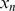 does not occur in foreground or background. The unweighted binary classification problem is a special case of this schema, where 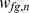 and 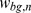 are either 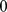 or 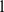, i.e., 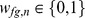 and 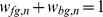.

### Confusion Matrix for Weighted and Unweighted Data

In Table 1, we present the schema of a confusion matrix for a binary classification problem. The entries of the confusion matrix are the numbers of true positives 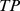, false positives 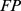, true negatives 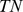, and false negatives 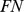. In addition, the table contains the number of foreground data points 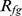, the number of background data points 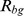, the number of foreground prediction 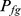, the number of background prediction 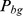, and the number of all data points 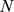.

**Table 1:** Binary confusion matrix.

		real label		
		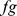	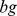	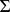
predicted label	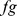	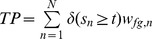	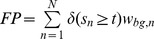	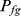
	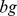	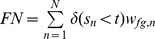	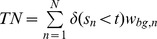	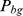
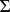		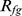	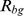	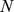

The confusion matrix can be computed for weighted and unweighted data. For unweighted data each data point contributes with a weight of one, whereas for weighted data each data point contributes with its specific weight for the given class.

Based on the classification score 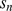 for data point 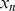 and a classification threshold 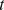, the entries can be computed as defined in Table 1. These numbers are integer values for unweighted data (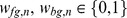), while they are non-negative real values for weighted data (Table 2).

**Table 2:** Classification for unweighted and weighted data.

(a) Classification scores, labels and weights					
classification score	class	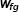	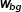		
2.54	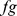	0.9	0.1		
2.37	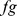	0.92	0.08		
1.56	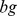	0.22	0.78		
1.35	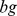	0.07	0.93		
0.06	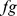	0.67	0.33		
−1.08	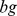	0.09	0.91		
**(b) Confusion matrices for unweighted and weighted data**					
	**unweighted**	****		**weighted**	
	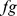	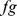		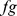	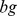
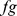	TP = 2	FP = 1		TP = 2.04	FP = 0.96
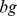	FN = 1	TN = 2		FN = 0.83	TN = 2.17

The entries of a confusion matrix have been calculated for a classification threshold of 1.5. In case of unweighted data, the class label is 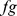 if 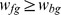 and otherwise 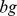.

Varying the classification threshold leads to a series of confusion matrices and corresponding performance measures that can be visualized by ROC and PR curves. However, previous interpolations for computing PR curves from a number of supporting points [7] are not applicable to weighted data as defined in the previous sub-section. In the following, we propose an alternative interpolation for computing PR curves, which is directly applicable to all types of weights considered.

### Discrete Interpolation of Precision-recall Curves for Unweighted Data

For unweighted data, Davis and Goadrich notice that it is usually not reasonable to use linear interpolation for the PR curve if two adjacent points of the PR curve differ by more than one true positive [7]. For this reason, they propose to use a piecewise linear interpolation along the true positives between the confusion matrices underlying these points. For reasons of simplicity, we follow Davis and Goadrich [7] and focus on the interpolation between two points 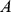 and 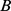 obtained from different classification thresholds with the corresponding numbers of true and false positives 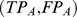 and 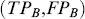 where 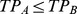 and, hence, 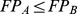.

The interpolation introduces intermediate points, which make linear interpolation more reasonable than the linear interpolation between the points 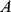 and 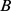. The interpolation between the original points 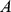 and 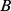 is described by
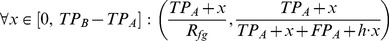
(1)for integer values of 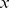 and with 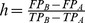. Using these intermediate points, the trapezoidal rule is used for computing the AUC-PR [7]. In the remainder of this manuscript, we denote this interpolation by *discrete-TP*.

One alternative to a stepwise interpolation along the true positives is a stepwise interpolation along the false positives, yielding
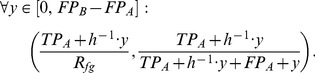
(2)


In analogy to the method of [7], the trapezoidal rule could be used for computing the AUC-PR. In the remainder of this manuscript, we denote this interpolation by *discrete-FP*.

### Continuous Interpolation of Precision-recall Curves for Weighted and Unweighted Data

Here, we propose a piecewise-defined function allowing to compute the AUC-PR by a sum of integrals. In general, we can compute the AUC-PR by parameterizing the PR curve by
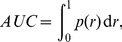
(3)where 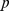 and 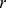 denote precision and recall, respectively [24]. Brodersen *et al.* use this parameterization in an 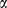-binormal model [24], which requires the assumption that the distribution of scores in both classes follows a normal distribution. In contrast, we propose a piecewise definition of the curve that requires no additional assumptions, which is closely related to the definition published by Boyd *et al.* [25] while this manuscript was in preparation.

We use a piecewise definition for computing the PR curve and the area under this curve. Specifically, we compute
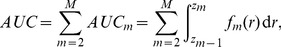
(4)where 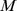 is the number of sorted intermediate recall values 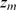, and 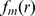 is one piece of the piecewise-defined function. Based on equation (1) and the substitution 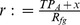, we propose to compute the AUC between 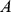 and 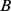 by



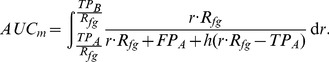
(5)This integral can be easily solved by substituting 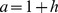 and 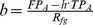, and subsequently rewriting the integrand of equation (5) as 
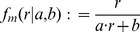
, yielding
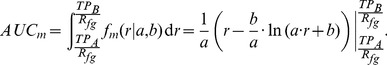
(6)


Equation (6) states how to compute the AUC between the two points 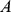 and 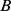. For computing the complete AUC-PR, we sum up the contributions between all adjacent points of the curve. In Table 3 (Algorithm 1), we provide pseudo code for computing the complete AUC-PR.

**Table 3:** Algorithm 1: Pseudo code for computing the AUC-PR based on the continuous interpolation.

	determine *A* with *TP_A_* = *R_fg_*			
	*auc* = 0			
	**while** *TP_A_* > 0 **do**			
		*B* = *A*		
		determine new *A*		
		**If** *TP_A_* < *TP_B_* then		
			determine *a*, *b*, *h*	
			*pB* = *TP_B_*/ *R_fg_*	
			*pA* = *TP_A_*/ *R_fg_*	
			**If** *b* ≠ 0 **then**	
				*auc* + = (*pB* – *pA* – *b*/ *a*⋅(log(*a⋅pB*+*b*) – log(*a⋅pA* + *b*)))/*a*
			**else**	
				*auc* + = (*pB* – *pA)*/*a*
			**end**	
		**end**		
**end**				

Initially, we choose the classification threshold such that the number of true positives is equal to the total number of positives. Then we iterate as long as the number of true positives – and, hence, recall – is greater than 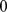. We determine the new point 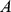 by choosing the next existing score as classification threshold. Unless this threshold leads to an identical number of true positives, we compute the values of 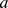, 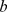, and 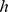 as defined by equation (6), and set the borders of the integration. We use these values to compute the AUC between the current points 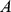 and 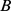, and proceed with the while-loop. After termination of the loop, 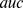 holds the AUC-PR.

The goal of this manuscript is the computation of PR curves and AUC-PR values for weighted data as introduced in section. In this case, the entries of the confusion matrix are accumulated weights of the corresponding data points (cf. Table 1) yielding a real-valued confusion matrix (cf. Table 2).

Discrete interpolations depend on the step size of the interpolation. In case of unweighted data, a step size of one is reasonable, because it corresponds to one data point. However, it is not obvious how to choose a reasonable step size for weighted data.

In contrast to discrete interpolations, the continuous interpolation based on equation (6) can be directly applied to real-valued confusion matrices. When we replace the integration limits of equation (6) by the corresponding entries of the real-valued confusion matrix, i.e., the ratios of the sums of the weights of true positive and all foreground data points, we obtain a definition of the AUC-PR for weighted data. Hence, the continuous interpolation allows the computation of the AUC-PR for weighted data.

### Characteristics of AUC-PR

Three characteristics are central for each performance measure: its maximum, its average for a random classifier, and its minimum. In the Text S1, we prove the following Theorem.

**Theorem 1** *Let* 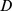 *be a weighted data set of* 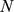 *data points and* 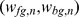 *be the weights for data point* 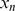 *. Furthermore, let* 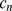 *be the classification score of data point* 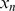 *assigned by a classifier, and let* 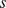 *be the order of classification scores, i.e.,*

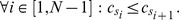

1. *The maximal AUC-PR is obtained iff the weights of the data points are monotonically increasing with respect to the sorting* 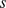, *i.e.*,

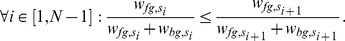

2. *The minimal AUC-PR is obtained iff the weights of the data points are monotonically decreasing with respect to the sorting* 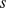, *i.e.*,

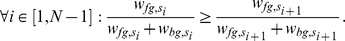



Based on the minimum and the maximum, normalized performance measures as for instance the normalized AUC-PR can be computed [26]. In the following, we study these three characteristics of the AUC-PR based on continuous interpolation for unweighted and weighted test data sets.

First, we consider the optimal classifier, i.e., the classifier with the maximal AUC-PR. For unweighted test data, the optimal classifier always yields an AUC-PR of 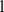. Hence, we focus on weighted data sets in the following. We reach a recall of 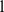 only if all data points are assigned to the foreground. In this case, the precision is computed as sum of all foreground weights divided by the sum of all foreground weights 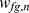 and corresponding background weights 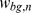. If at least one data point has a foreground and background weight greater than 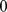, i.e., if 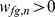 and 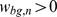 then the sum of the corresponding 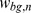 is greater than 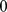, and a precision of 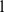 can not be gained. For this reason, it is impossible to reach a precision of 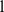 for a recall of 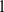 and, hence, an AUC-PR of 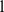 for weighted data.

**Figure 1:**
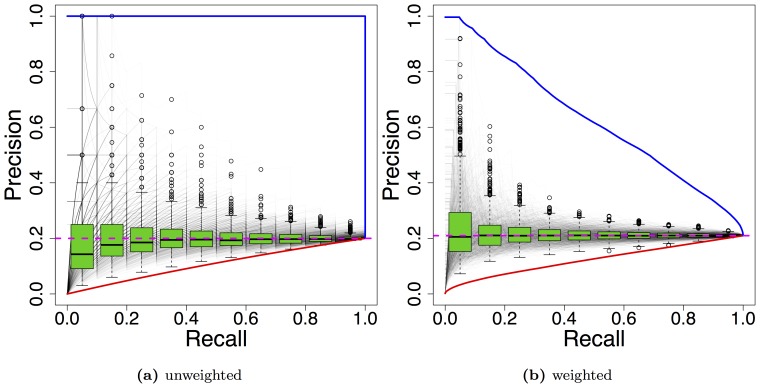
Precision recall curves for data set with 100 data points and class ratio 1 to 4. The blue and the red curve indicate estimators of the best and the worst curve, respectively. The gray curves represent 1,000 PR curves based on a random scored-based classifications, which are also summarized by the green boxplots. The pink dashed line indicates the level of the class ratio 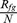.

In Figure 1, the blue PR curves yield the maximal AUC-PR according to Theorem 1. In Figure 1(a), the AUC-PR of the optimal classifier is equal to 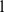. In contrast, Figure 1(b) clearly indicates that the point 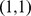 cannot be reached leading to an AUC-PR smaller than 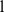. Accordingly, the maximal value of AUC-PR depends on the weights and we cannot provide a closed-form solution of this maximal value.

Second, we consider the worst classifier, i.e., the classifier with the minimal AUC-PR. Such a classifier always decides for the class with the lower confidence. In Figure 1, the red PR curves yield the minimal AUC-PR according to Theorem 1. Trivially, the minimal AUC-PR is larger than 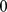 in both cases. The worst possible classifier initially yields a very low precision, since the first data points classified as foreground always have a greater background weight 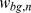 than foreground weight 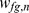. With increasing recall, the precision increases as well, because additional data points with increasing foreground weight and, hence, decreasing background weight are added to the foreground predictions. Finally, the worst possible classifier reaches the common point of all classifiers with a recall of 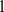 and a precision of 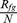, i.e., the sum of all foreground weights 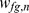 divided by the sum of all foreground and all background weights.

Finally, we consider the AUC-PR for random guessing. Since random guessing can not be represented by a single random classifier, we investigate an ensemble of 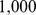 random classifiers. In Figure 1, we illustrate the corresponding curves in gray. To give a better overview, we summarize them by green boxplots. By inspecting the characteristics of these curves, we find in both cases that the median of the boxplots is approximately the class ratio 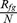. Hence for weighted data, in analogy to the case of unweighted data, a random classifier yields an AUC-PR of approximately the class ratio.

## Results and Discussion

In this section, we investigate (i) theoretical and practical differences between the discrete and continuous interpolations and (ii) whether the extension of PR and ROC curves to weighted data may possibly allow a more detailed classifier assessment.

### Theoretical Comparison of Discrete and Continuous Interpolations of the Precision-recall Curve

First, we investigate in which situations the discrete and the continuous interpolations yield identical segments of the curve and, hence, identical contributions to the AUC. If a segment of the continuous interpolation is linear, it is identical to the discrete interpolations. We obtain linear segments in two situations. On the one hand, we obtain a vertical linear segment if 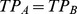, which means that the number of true positives and, hence, the recall is equal for points 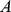 and 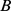. On the other hand, we obtain a horizontal segment if the function 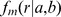 is constant. We obtain a constant function if 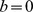, which is the case if the ratio of true positives and false positives is identical at both points (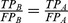) or, equivalently, if the ratio of the true positives at the two points is equal to the ratio of the false positives (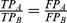).

**Figure 2:**
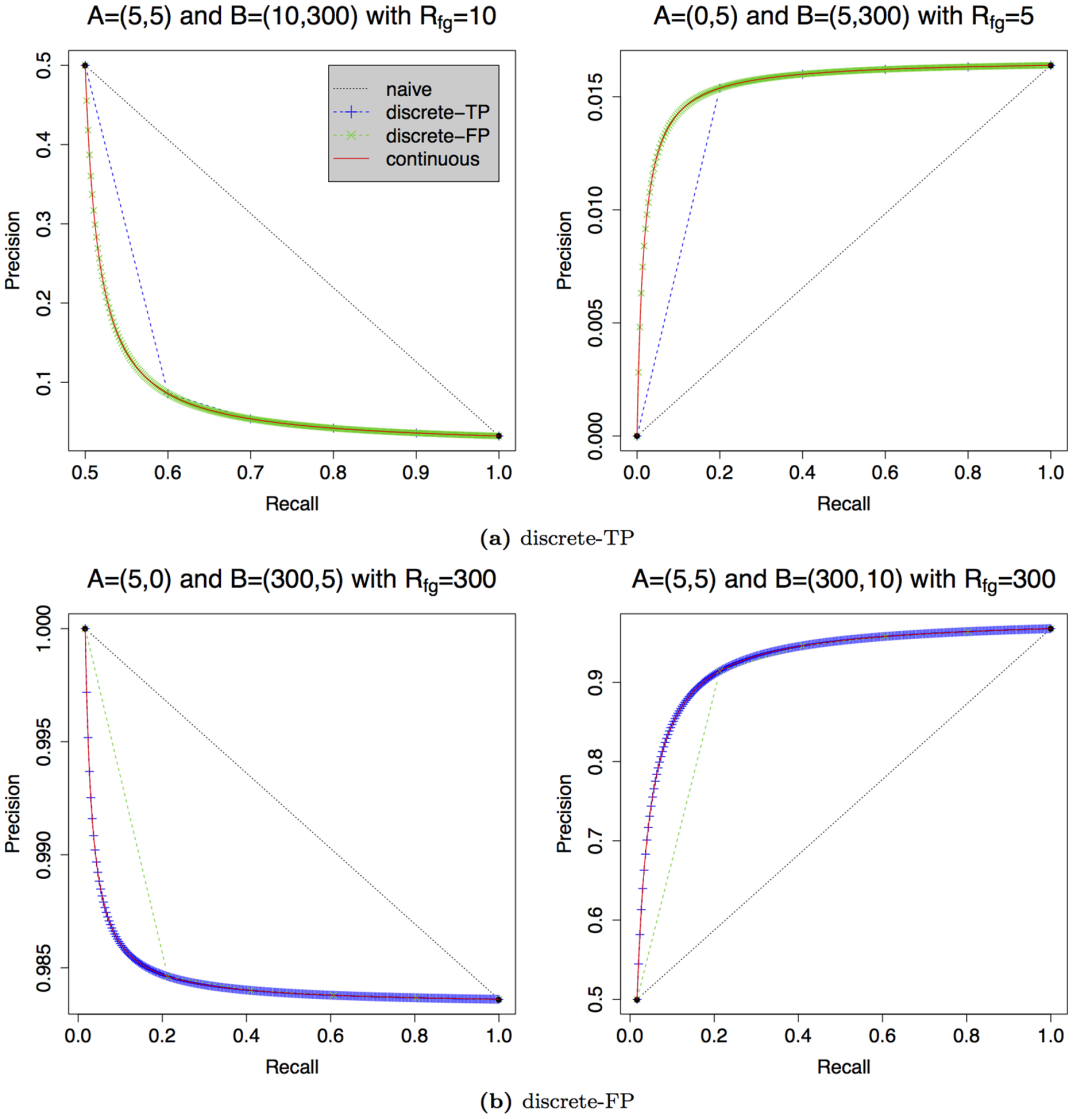
Differences between discrete and the continuous interpolations of the PR curve. The figure shows for each discrete interpolation (along true positives or along false positives) one example of a larger and smaller AUC between two supporting points.

Second, we investigate situations that result in a large deviation between discrete and continuous interpolation. Such situations occur if the discrete interpolations span a large range with few intermediate points. In Figure 2, we show examples where the continuous interpolation results in a substantially larger or smaller AUC-PR than one of the discrete interpolations.

In Figure 2(a), we consider cases exhibiting large deviations between the discrete interpolation discrete-TP and the continuous interpolation. For both cases, the points 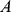 and 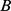 differ by only 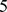 true positives, which leads to a coarse interpolation with a lower number of intermediate points for the interpolation along the true positives. Since the differences of false positives are substantially greater (295) than the number of true positives (5) for both plots, the interpolation discrete-FP is much more fine grained. In both cases, the continuous interpolation plotted as a red curve contains all intermediate points of both interpolations, and the AUC-PR is almost identical to that of the interpolation discrete-FP.

In Figure 2(b), we consider the opposite case exhibiting large deviations between the discrete interpolation discrete-FP and the continuous interpolation. Here, the difference of true positives between the points 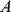 and 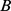 is large, whereas the difference of false positives is small. For this reason, the interpolation discrete-TP becomes fine grained, whereas the interpolation discrete-FP uses only a low number of intermediate points. Again, the continuous interpolation includes all intermediate points of both discrete interpolations, and the AUC-PR is almost identical to that of the interpolation discrete-TP.

In summary, we find that the continuous interpolation fits the discrete interpolation with more intermediate points in all four cases.

### Practical Comparison of Discrete and Continuous Interpolations of the Precision-recall Curve

In this section, we compare the interpolations for complete curves. To this end, we sample classification scores at a fixed class ratio of 1 to 10 for foreground versus background. Analyzing the AUC-PR for the interpolation discrete- TP and the continuous interpolation, we vary the size of the foreground data set as well as the uniqueness of the classification scores. To achieve the latter, we sample the classification scores from different numbers of bins.

**Figure 3:**
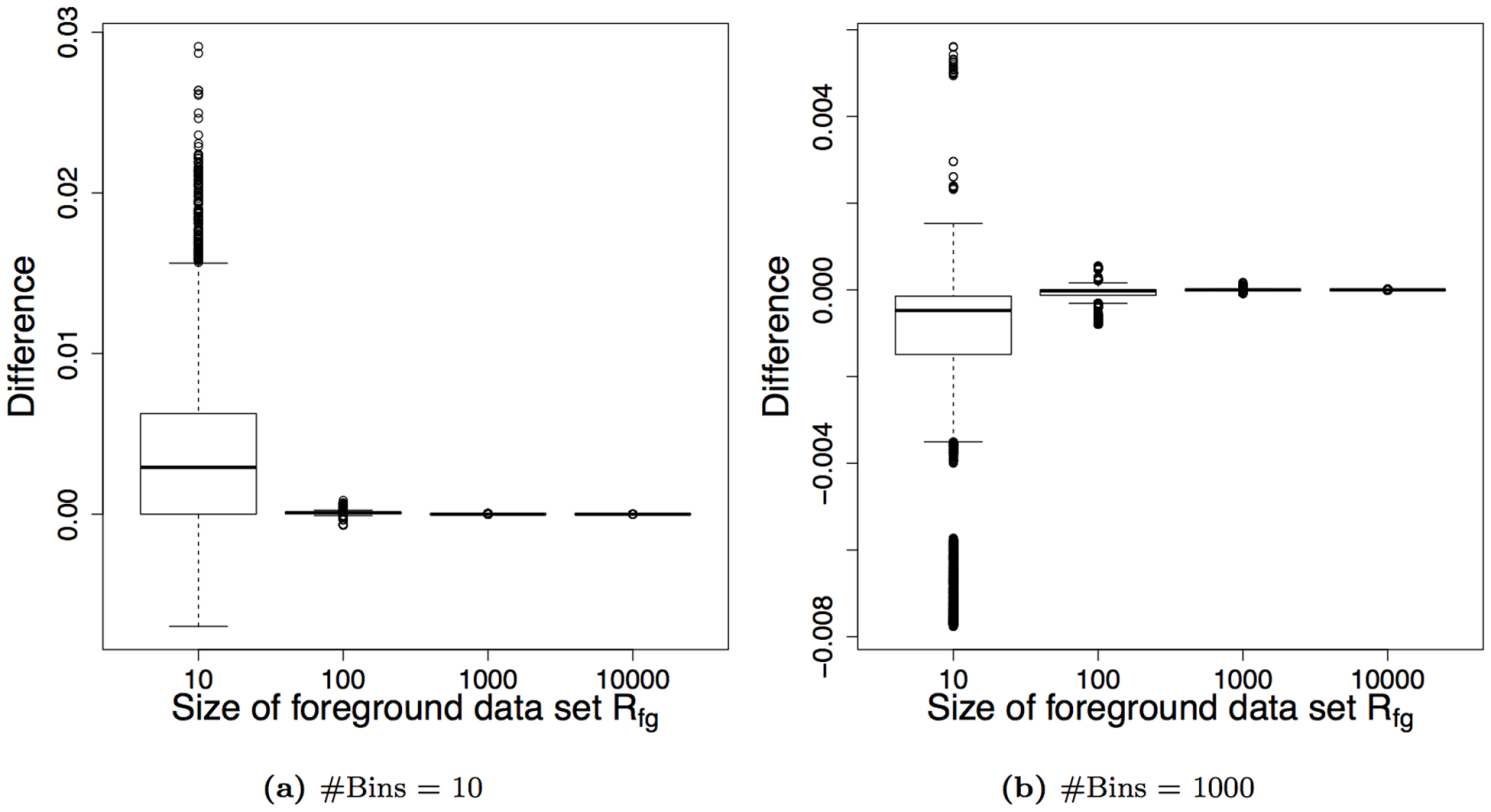
Differences of AUC-PR between the interpolations for varying size of the foreground data set. Panel (a) depicts the results for 10 bins equivalent to at most 10 different classification scores, whereas panel (b) depicts the results for 1,000 bins.

Trying to obtain almost equally distributed AUC-PRs, we sample the classification scores from a normal distribution with mean normally distributed around 1.64 for the foreground and fixed mean 0 for the background. (The value 1.64 is based on the class ratio of 1 to 10 and the quantile function, 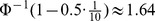.) In each case we use a standard deviation of 1. In Figure 3, we present the results of this simulation repeating the above-described procedure 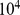 times.

At a first glance, we observe that the difference between the two interpolations can be up to 0.03. We find this difference for the smallest data sets comprising 10 and 100 test data points for foreground and background, respectively, and for the least number of unique classification scores with at most ten different values. However, 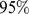 of the absolute differences are smaller than approximately 0.01.

In addition, increasing the size of the data sets or the number of bins, we find that the difference between both interpolations reduces drastically to almost 0. Both findings can be explained by a greater number of supporting and intermediate points and, hence, a more fine-grained coverage along the recall leading to converging AUC-PR values for continuous and discrete-TP interpolation. Hence, the discrete-TP and the continuous interpolation are similarly good approximations. This means that conclusions drawn from the interpolation discrete-TP for unweighted data are usually also valid for the proposed continuous interpolation and vice versa. However, the continuous interpolation allows to directly compute the PR curve and the AUC-PR for weighted data.

### Comparison of Weighted and Unweighted Test Data

In this section, we illustrate the main benefit of the proposed continuous interpolation, which is its applicability to weighted data. More specifically, we show in a simulation study that classifiers that yield an indistinguishable performance using unweighted test data may indeed achieve a considerably different performance using weighted test data. Inspecting the relationship of the classification scores of these classifiers to the given weights, we show that the ranking of classifiers using ROC and PR curves for weighted test data is reasonable.

**Figure 4:**
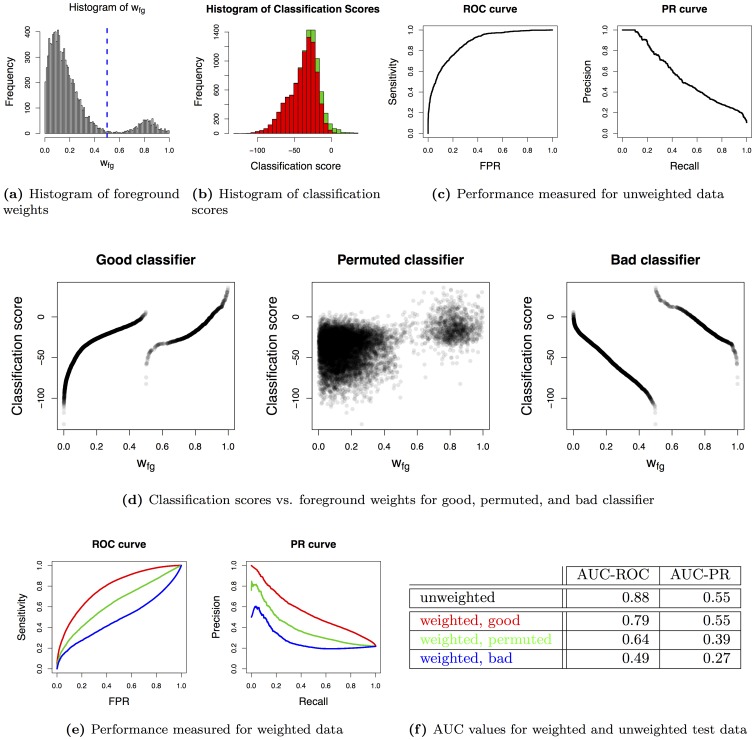
PR and ROC curves and respective AUC values for weighted and unweighted data. Panel (a) show a histogram of foreground weights (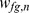) for all data points. The dashed line indicates the threshold used to separate foreground and background data points in the unweighted case. Panel (b) presents a histogram of classification scores. Within the bars of the histogram, we visualize the number of data points from the foreground (green) and background (red) class according to the unweighted case. Panel (c) presents classification performance using unweighted data computed from the classification scores presented in panel (b). Panel (d) visualizes the relationship between classification scores and weights for the hypothetical good, permuted, and bad classifiers. All three orderings of classification scores share the same underlying distribution as shown in panel (b). Panel (e) show the clearly distinguishable classification performance of the three classifiers as measured by ROC and PR curves using weighted data. The corresponding AUC values are listed in panel (f).

To this end, we generate simulated data as follows: We first sample weights (i.e., 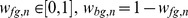) from a mixture of beta distributions for 10 000 hypothetical data points 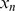 as presented in Figure 4(a). In addition to these weights, we obtain hard class labels by assigning all data points with 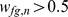 to the foreground class and all data points with 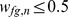 to the background class, yielding a class ratio of approximately 1∶9.

We further sample 10 000 classification scores, one for each data point assigned to the positive class and one for each data point assigned to the negative class according to the unweighted case as shown in Figure 4(b). For computing ROC and PR curves for unweighted test data (Figure 4(c)), no additional information is needed. In particular, the values of 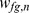 and 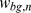 are neglected given the hard-labeling into foreground and background data points.

We generate three different hypothetical classifiers by different assignments of classification scores to the data points within each class as visualized in Figure 4(d). We conduct this assignment separately for the data points of each class (according to the unweighted case) to yield an identical classification performance for all three classifiers when using ROC and PR curves for unweighted test data.

We create a *good* classifier by assigning classification scores to the data points of each class in the order given by the foreground weights 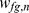, i.e., the data point with the smallest 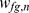 also obtains the smallest classification score, and the data point with the largest 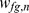 obtains the largest classification score. Notably, this is not the optimal classifier according to Theorem 1, because the classification scores in both classes are sorted separately.

We create a *permuted* classifier by assigning classification scores to the data points of each class in random order.

We further create a *bad* classifier by assigning classification scores to the data points of each class in the reverse order compared to the foreground weights 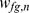, i.e., the data point with the smallest 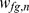 obtains the largest classification score, and the data point with the largest 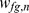 obtains the smallest classification score. Again, this is not the worst possible classifier according to Theorem 1, because the classification scores in both classes are sorted separately. However, the good and the bad classifier yield the maximal and minimal AUC-PR under this additional constraint, respectively.

Due to this setup, the distribution of classification scores within each class according to the unweighted case is identical and, hence, all three classifiers obtain identical ROC and PR curves for unweighted data depicted in Figure 4(c).

However, if we consider the relationship of classification scores and weights as shown in Figure 4(d), we might intuitively favor the good classifier over the permuted and even more over the bad classifier. This intuitive ranking is reflected by the ROC and PR curves that are computed using weighted test data (Figure 4(e)), where the curves of the good classifier consistently lie above the curves of the permuted classifier and the curves of the permuted classifier consistently lie above those of the bad classifier.

A similar picture emerges for the areas under the ROC and PR curves as listed in Figure 4(f). Using unweighted test data, all three classifiers yield an identical AUC-ROC and an identical AUC-PR as given in the first line. Using weighted test data, however, the three classifiers obtain AUC-ROC and AUC-PR values that correspond to the intuitive ranking of classifiers.

Hence, we may conclude that the applicability of PR curves to weighted test data, which has been achieved by the interpolation of equation (6), adds a new dimension to the assessment of classifiers. This additional dimension allows us to distinguish classifiers by their performance that would be indistinguishable by traditional PR curves neglecting existing weights for test data.

This example also illustrates that the transition from unweighted to weighted data for computing ROC and PR curves changes the objective measured by these curves. While traditional ROC and PR curves using unweighted test data only consider the distribution of classification scores within the two classes, ROC and PR curves using weighted test data additionally take into account the confidence of the labeling. Hence, these curves measure the ability of a classifier to reconstruct the ordering of data points according to the weighted 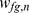, which makes them suitable performance measures for regression problems as well. Nonetheless, ROC and PR curves using weighted test data do not make assumptions about a functional relationship between classification scores and weights as it would be the case, for instance, using Pearson correlation as performance measure.

### Practical Application

In this section, we evaluate the efficacy of AUC-PR for weighted data in practical applications. To this end, we compare the rankings of classifiers based on their AUC-PR for weighted and unweighted data in in two real-world examples.

**Figure 5:**
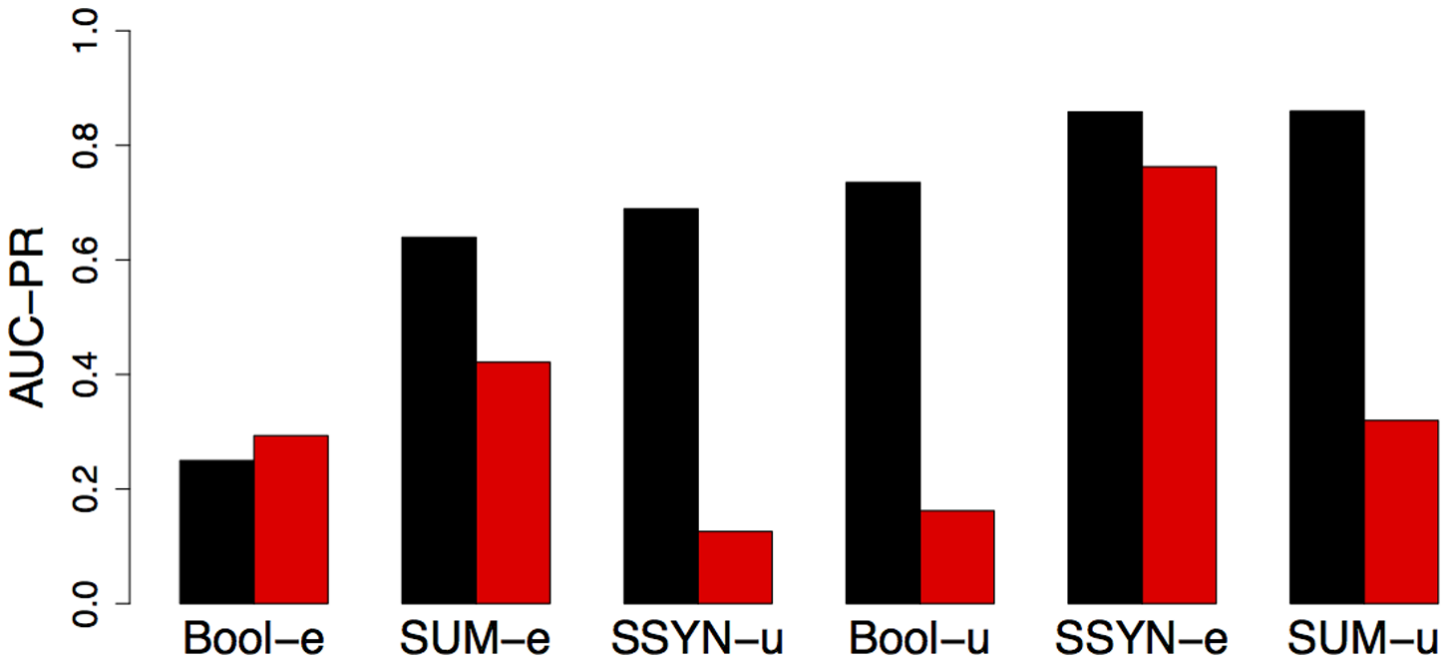
Comparison of ranking classifiers by AUC-PR using unweighted and weighted test data for query 29 from [18]. The AUC-PR for unweighted test data is depicted in black, whereas the AUC-PR for weighted test data is depicted in red.

In Figure 5, we present a reassessment of classifiers from an information retrieval task [18]. The authors kindly provided the data weights and classifier scores (query 29), and we use these scores to re-evaluate the classifiers in terms of AUC-PR for weighted and unweighted test data. Interestingly, we find different rankings of the six classifiers based on weighted and unweighted test data. For unweighted test data SSYN-e and SUM-u perform comparably, whereas for weighted test data SSYN-e clearly outperforms SUM-u. The classifiers SSYN-u and Bool-u perform moderately for unweighted data, but perform worst for weighted test data. In contrast, the classifier Bool-e, which performs worst for unweighted data, yields a performance similar to that of SUM-u for weighted data, which clearly outperforms Bool-e for unweighted test data. These different rankings of the classifiers show that neglecting weights of test data might lead to different and possibly erroneous conclusions.

In a second case study, we perform a reassessment of classifiers from bioinformatics [27] evaluating the performance of classifiers for 66 protein binding microarray (PBM) data sets. PBMs measure the *in-vitro* binding affinity of transcription factors to DNA sequences using microarrays in an unbiased manner, where double-stranded probe sequences are chosen such that they contain all 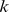-mers up to a given 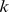 with identical frequency. The goal of that study was to assess different classifiers for their ability to distinguish bound from unbound probes, and for the correspondence of their classification scores (e.g., likelihood ratios) to measured microarray intensity values.

In the original publication [27], the authors introduce an unweighted labeling based on the intensity values for all probes sequences in each of the 66 experiments. For each individual experiment, they define the threshold separating foreground and background data points as the mean intensity values plus four times the standard deviation of intensity values, but requiring at least 50 foreground data points. Based on this labeling, they compare classifiers for instance based on the mean AUC-ROC over all experiments.

**Figure 6:**
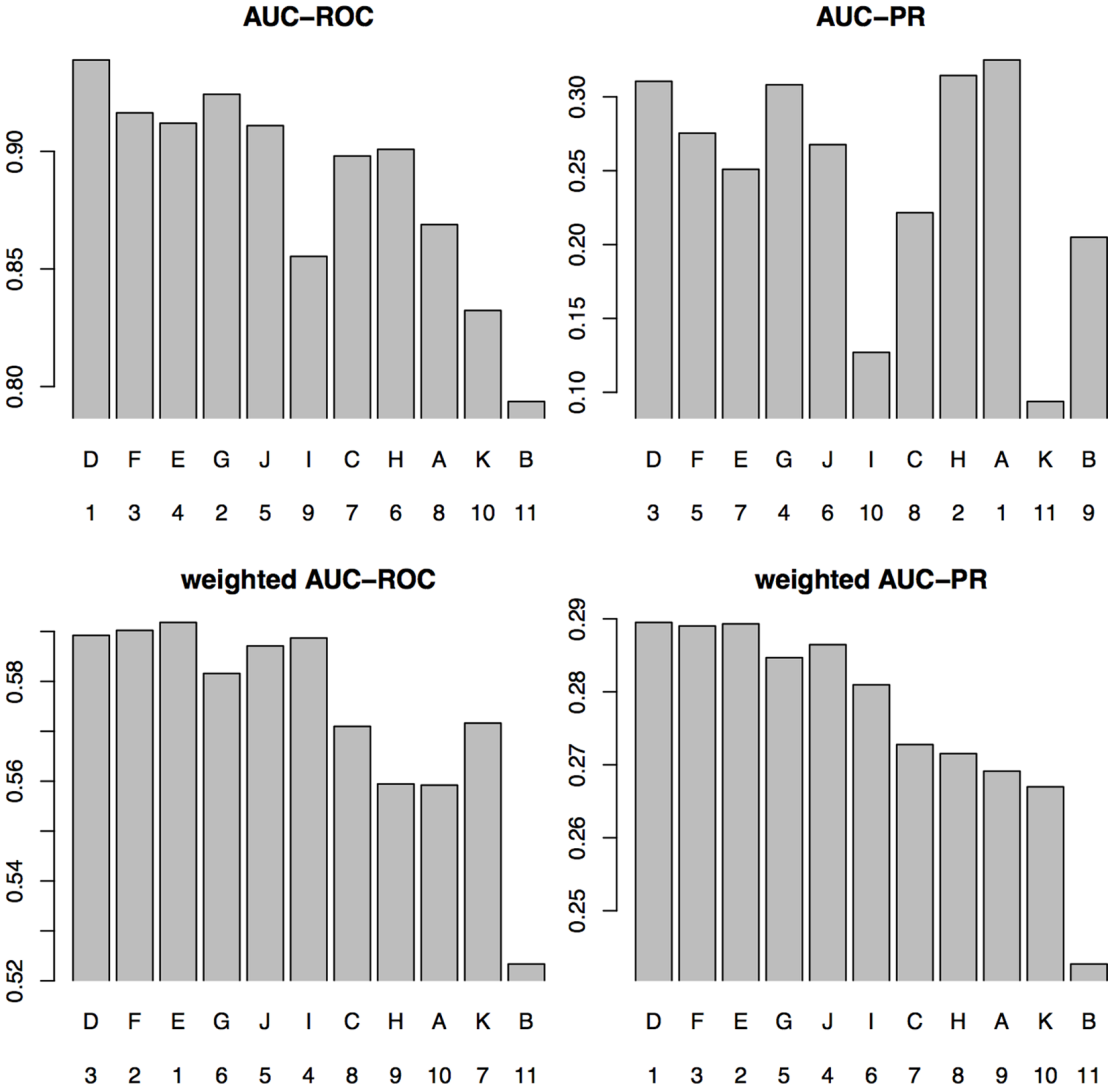
Mean results for AUC-ROC and AUC-PR on PBM data sets using unweighted or weighted test data. The team name and the ranking is depicted on the abscissa, while the mean result for AUC-ROC and AUC-PR is depicted on the ordinate. Teams are displayed in the order of the original ranking of Weirauch *et al.* [27].

**Figure 7:**
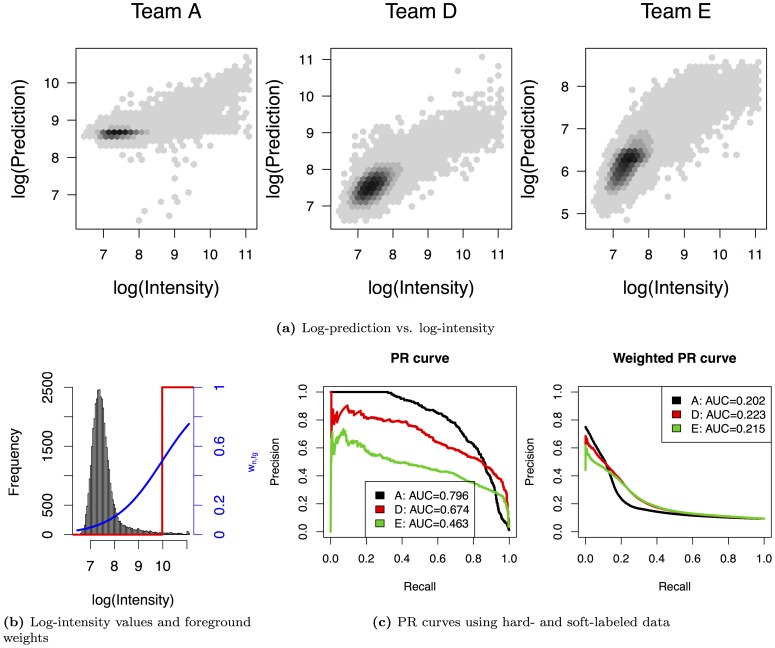
Comparison of PR curves using unweighted and weighted test data for one exemplary data set (11) of [27]. In panel (a), we plot the predicted log-intensity values of classifiers A, D, and E against the measured log-intensity values. Panel (b) visualizes the class border in the unweighted case (red line) and the weights of the foreground class (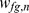) in the weighted case. In panel (c), we show the PR curves of the three classifiers using unweighted (left) and weighted (right) test data.

In Figure 6, we compare the mean AUC-ROC, the mean AUC-PR, and the corresponding rankings for unweighted and weighted test data. In the unweighted case, we take the class labels suggested by Weirauch *et al.* [27]. In the weighted case, we use a logistic function with slope 1 that is shifted along the intensity values such that a foreground weight of 0.5 is obtained at the threshold of the unweighted case (cf. Figure 7(b)).

We find that the rankings for both mean AUC-ROC and mean AUC-PR change considerably going from unweighted to weighted test data. Focusing on the mean AUC-PR, we find that the ranking obtained by AUC-PR using weighted test data are in better accordance to the original ranking of Weirauch *et al.* than the ranking using unweighted test data.

Three classifiers with exceptionally different rankings are A, D, and E, which obtain ranks 1, 3, and 7 considering unweighted test data, and ranks 9, 1, and 2 using weighted test data, respectively. Hence, we further investigate AUC-PR and PR-curves for classifiers A, D, and E for one exemplary data set (data set 11) in Figure 7. In Figure 7(a), we plot the predicted log-intensities of each of the three classifiers against the measured log-intensities for all probe sequences considered in this PBM experiment. Intuitively, we might favour classifiers D and E over classifier A, because their predictions appear to be in better accordance to the measured intensities.

In Figure 7(b), we present a histogram of the measured log-intensities for this data set. In addition, we illustrate the border between the foreground and the background for the unweighted case by a red line, and the foreground weights (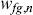) obtained by the logistic function as blue line. As explained above, we obtain 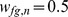 at the class border.

The PR curves of the three classifiers using weighted and unweighted test data are shown in Figure 7(c). In the unweighted case, classifier A yields a greater precision than classifier D, and classifier D yields a greater precision than classifier E for a wide range of recall values. This ranking is preserved in the weighted case only for a small range of low recall values, whereas for larger recall values, the greater precision is gained by classifiers D and E. The reason for this observation is that in the weighted case almost all data points (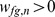) need to be assigned to the foreground by a classifier to yield a precision of 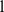. Preferably, these data points should receive classification scores with the same ordering as these weights (c.f. section), which is neglected using hard-labeling based on some threshold.

Thresholds or other rules for labeling data points are often chosen arbitrarily, and mildly different choices could often be justified no worse than the selected one. For this reason, we consider the stability of performance measures to mild changes of the class border an important property.

Hence, we finally investigate the stability of AUC-PR for unweighted and weighted test data using different thresholds for the labeling. To this end, we compute the mean AUC-PR using a threshold of mean intensity plus one standard deviation, and compare the results with those for the above-mentioned threshold of mean intensity plus four times standard deviation for all 11 classifiers considered in Figure 6.

**Figure 8:**
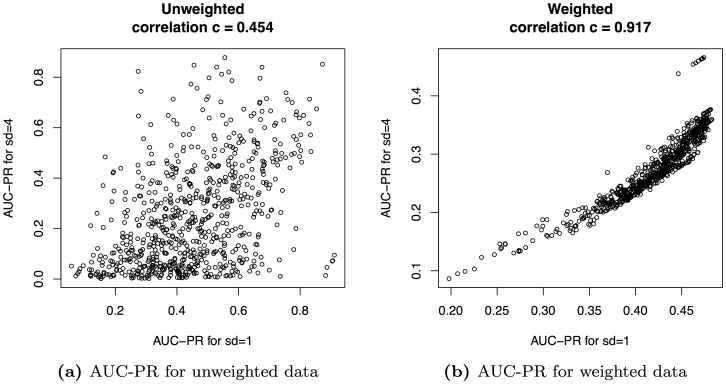
Comparison of AUC-PR values for different classification thresholds. In panel (a), we consider unweighted test data and plot the AUC-PR values for a threshold of mean intensity plus four times standard deviation (ordinate) against the AUC-PR values for a threshold of mean intensity plus four times standard deviation (abscissa). In panel (b), we consider weighted test data and plot the AUC-PR values in analogy to panel (a). We find a substantially greater Pearson correlation between the AUC-PR values for the different thresholds for weighted data compared to unweighted data.

We measure the stability of the assessments by the Pearson correlation of the AUC-PR values obtained for each of the 11 teams and each of the 66 data sets using either of the two thresholds. We present the results of this analysis in Figure 8. We find that AUC-PR for unweighted data yields a correlation coefficient of 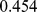 between the AUC-PR values for these two thresholds considering all 11 classifiers and all 66 data sets. In contrast, AUC-PR for weighted data yields a correlation coefficient of 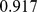. We obtain similar results using Spearman rank correlation (unweighted: 0.447; weighted: 0.967 ).

This indicates that the AUC-PR for appropriately weighted data is more stable leading to less changes in the ranking than the mean AUC-PR for unweighted data. Hence, the choice of the classification threshold, which is somewhat arbitrary, is down-weighted.

## Conclusions

PR curves and the areas under these curves have gained increasing importance in machine learning during the last years. Computing the area under the precision recall curve depends on the interpolation between adjacent points for given confusion matrices.

Here, we introduced a continuous interpolation for computing the area under the precision recall curve. We compared discrete and continuous interpolations theoretically and practically and show the interpolations are in agreement using unweighted data.

The continuous interpolation can also be used for weighted data sets. The optimal AUC-PR is not necessarily equal to 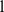 for weighted, so we derived conditions for gaining the maximum and minimum AUC-PR.

Based on artificial and real-world data sets, we found that the ranking of classifiers based on their AUC-PR might differ severely using unweighted and weighted test data sets. We also found that AUC-PR using weighted test data is less sensitive to small changes in the class border than AUC-PR using hard labels.

We implemented Table 3 (Algorithm 1) in the open-source Java library Jstacs [28] and make it publicly available with version 2.1. We provide a command line application for computing PR curves and AUC-PR from weighted and unweighted data at http://www.jstacs.de/index.php/AUC-PR.

## Supporting Information

Text S1 **The supplementary text contains all lemmata and proofs for proving Theorem 1.**
(PDF)
