# Supplementary material for: Area under Precision-Recall Curves for Weighted and Unweighted Data
Source: PLoS One. 2014 Mar 20;9(3):e92209. doi: 10.1371/journal.pone.0092209 (PMC3961324; doi:10.1371/journal.pone.0092209)
Supplement: Text S1 — The supplementary text contains all lemmata and proofs for proving Theorem 1. (PDF) [file pone.0092209.s001.pdf]

Text S1

In this appendix we prove Theorem 1 on the minimum and the maximum of the AUC-PR for weighted data. The idea of the proof is visualized in Figure S1 and can be used to follow the Lemmata and Theorem.

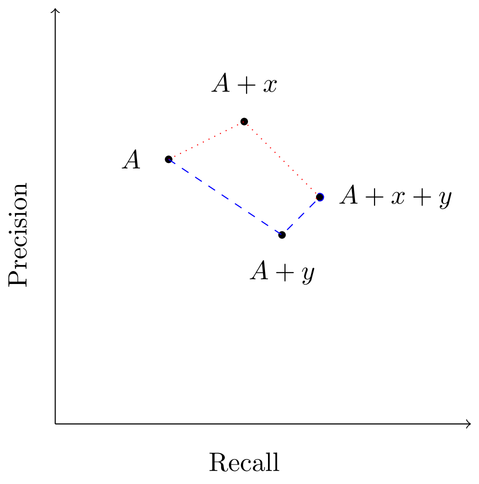

Figure S1. Idea of the proof of minimal and maximal AUC-PR. We consider the computation of a part of the PR curve starting from point A (with fixed TP_A and FP_A) and ending at point $A + x + y$ (with fixed $TP_A + w_{fg,x} + w_{fg,y}$ and $FP_A + w_{bg,x} + w_{bg,y}$). Between these two points, the PR curve can take two different ways, either using the intermediate point $A + x$ or using the intermediate point $A + y$. In the proofs, we show that depending on the weights of the data points x and y, one of these ways yields a greater partial AUC-PR than the other. Finally, we show that this result can be used to derive the maximum and minimum of the AUC-PR for weighted data in general.

Lemma 1. *Let* $A = \left(\frac{TP_A}{R_{fg}}, \frac{TP_A}{TP_A+FP_A}\right)$ *be a point on the PR-curve and* x *be the next data point to be classified with weight* $(w_{fg,x}, w_{bg,x})$*, then the contribution of* x *to the AUC-PR is:*

$$AUC_{A\to(A+x)} = \frac{w_{fg,x}}{w_{fg,x}+w_{bg,x}} \frac{1}{R_{fg}} \left[w_{fg,x} - \frac{w_{fg,x}\cdot FP_A - w_{bg,x}\cdot TP_A}{w_{fg,x}+w_{bg,x}} \cdot \ln\left(1 + \frac{w_{fg,x}+w_{bg,x}}{TP_A+FP_A}\right)\right]. \quad (1)$$

Proof. Based on equation (6), we can compute $h_{A\to(A+x)} = \frac{w_{bg,x}}{w_{fg,x}}$ and

$$\begin{aligned} AUC_{A\to(A+x)} = &\frac{1}{1+\frac{w_{bg,x}}{w_{fg,x}}} \frac{1}{R_{fg}} \Bigg[TP_A + w_{fg,x} - TP_A \\ &- \frac{FP_A - \frac{w_{bg,x}}{w_{fg,x}}\cdot TP_A}{1+\frac{w_{bg,x}}{w_{fg,x}}} \cdot \ln\left(\frac{TP_A + w_{fg,x} + \frac{w_{bg,x}}{w_{fg,x}}\cdot(TP_A + w_{fg,x} - TP_A) + FP_A}{R_{fg}}\right) \\ &+ \frac{FP_A - \frac{w_{bg,x}}{w_{fg,x}}\cdot TP_A}{1+\frac{w_{bg,x}}{w_{fg,x}}} \cdot \ln\left(\frac{TP_A + \frac{w_{bg,x}}{w_{fg,x}}\cdot(TP_A - TP_A) + FP_A}{R_{fg}}\right)\Bigg] \\ = &\frac{w_{fg,x}}{w_{fg,x}+w_{bg,x}} \frac{1}{R_{fg}} \Bigg[w_{fg,x} - \frac{w_{fg,x}\cdot FP_A - w_{bg,x}\cdot TP_A}{w_{fg,x}+w_{bg,x}} \\ &\cdot \left(\ln\left(\frac{TP_A + w_{fg,x} + w_{bg,x} + FP_A}{R_{fg}}\right) - \ln\left(\frac{TP_A+FP_A}{R_{fg}}\right)\right)\Bigg] \end{aligned}$$

$$= \frac{w_{fg,x}}{w_{fg,x} + w_{bg,x}} \frac{1}{R_{fg}} \left[w_{fg,x} - \frac{w_{fg,x} \cdot FP_A - w_{bg,x} \cdot TP_A}{w_{fg,x} + w_{bg,x}} \cdot \ln \left(1 + \frac{w_{fg,x} + w_{bg,x}}{TP_A + FP_A} \right) \right].$$

□

Lemma 2. *Let* $A = \left(\frac{TP_A}{R_{fg}}, \frac{TP_A}{TP_A + FP_A} \right)$ *be a point on the PR-curve. Furthermore, let* x *and* y *be the next two data points (in that order) with weights* $(w_{fg,x}, w_{bg,x})$ *and* $(w_{fg,y}, w_{bg,y})$*, respectively, then the contribution of* x *and* y *to the AUC-PR is:*

$$AUC_{A \to (A+x) \to (A+x+y)} = \frac{1}{R_{fg}} \left(\frac{w_{fg,x}}{w_{fg,x} + w_{bg,x}} \left[w_{fg,x} - \frac{w_{fg,x} \cdot FP_A - w_{bg,x} \cdot TP_A}{w_{fg,x} + w_{bg,x}} \cdot \ln \left(1 + \frac{w_{fg,x} + w_{bg,x}}{TP_A + FP_A} \right) \right] \right.$$
$$+ \frac{w_{fg,y}}{w_{fg,y} + w_{bg,y}} \left[w_{fg,y} - \left(\frac{w_{fg,y} \cdot FP_A - w_{bg,y} \cdot TP_A}{w_{fg,y} + w_{bg,y}} + \frac{w_{fg,y} \cdot w_{bg,x} - w_{bg,y} \cdot w_{fg,x}}{w_{fg,y} + w_{bg,y}} \right) \right.$$
$$\left. \left. \cdot \ln \left(1 + \frac{w_{fg,y} + w_{bg,y}}{TP_A + w_{fg,x} + FP_A + w_{bg,x}} \right) \right] \right) \tag{2}$$

Proof. Based on Lemma 1, we compute

$$AUC_{A \to (A+x) \to (A+x+y)} = AUC_{A \to (A+x)} + AUC_{(A+x) \to (A+x+y)}$$
$$= \frac{w_{fg,x}}{w_{fg,x} + w_{bg,x}} \frac{1}{R_{fg}} \left[w_{fg,x} - \frac{w_{fg,x} \cdot FP_A - w_{bg,x} \cdot TP_A}{w_{fg,x} + w_{bg,x}} \cdot \ln \left(1 + \frac{w_{fg,x} + w_{bg,x}}{TP_A + FP_A} \right) \right]$$
$$+ \frac{w_{fg,y}}{w_{fg,y} + w_{bg,y}} \frac{1}{R_{fg}} \left[w_{fg,y} - \frac{w_{fg,y} \cdot (FP_A + w_{bg,x}) - w_{bg,y} \cdot (TP_A + w_{fg,x})}{w_{fg,y} + w_{bg,y}} \right.$$
$$\left. \cdot \ln \left(1 + \frac{w_{fg,y} + w_{bg,y}}{TP_A + w_{fg,x} + FP_A + w_{bg,x}} \right) \right]$$
$$= \frac{1}{R_{fg}} \left(\frac{w_{fg,x}}{w_{fg,x} + w_{bg,x}} \left[w_{fg,x} - \frac{w_{fg,x} \cdot FP_A - w_{bg,x} \cdot TP_A}{w_{fg,x} + w_{bg,x}} \cdot \ln \left(1 + \frac{w_{fg,x} + w_{bg,x}}{TP_A + FP_A} \right) \right] \right.$$
$$+ \frac{w_{fg,y}}{w_{fg,y} + w_{bg,y}} \left[w_{fg,y} - \left(\frac{w_{fg,y} \cdot FP_A - w_{bg,y} \cdot TP_A}{w_{fg,y} + w_{bg,y}} + \frac{w_{fg,y} \cdot w_{bg,x} - w_{bg,y} \cdot w_{fg,x}}{w_{fg,y} + w_{bg,y}} \right) \right.$$
$$\left. \left. \cdot \ln \left(1 + \frac{w_{fg,y} + w_{bg,y}}{TP_A + w_{fg,x} + FP_A + w_{bg,x}} \right) \right] \right)$$

□

Lemma 3. *Let* $A = \left(\frac{TP_A}{R_{fg}}, \frac{TP_A}{TP_A + FP_A} \right)$ *be a point on the PR-curve. Furthermore, let* x *and* y *be the next two data points to be classified with weights* $(w_{fg,x}, w_{bg,x})$ *and* $(w_{fg,y}, w_{bg,y})$*, respectively. If* $\frac{w_{fg,x}}{w_{fg,x} + w_{bg,x}} > \frac{w_{fg,y}}{w_{fg,y} + w_{bg,y}}$*, then*

$$AUC_{A \to (A+x) \to (A+x+y)} > AUC_{A \to (A+y) \to (A+x+y)} \tag{3}$$

Proof. Based on Lemma 2 we compute the difference between both options. For reasons of simplicity we multiply by the constant R_{fg}.

$$
\begin{aligned}
\Delta &= R_{fg}\left(AUC_{A\to(A+x)\to B} - AUC_{A\to(A+y)\to B}\right)\\
&= \frac{w_{fg,x}}{w_{fg,x}+w_{bg,x}}\frac{w_{fg,x}\cdot FP_A - w_{bg,x}\cdot TP_A}{w_{fg,x}+w_{bg,x}}\left(\ln\left(1+\frac{w_{fg,x}+w_{bg,x}}{TP_A+w_{fg,y}+FP_A+w_{bg,y}}\right)-\ln\left(1+\frac{w_{fg,x}+w_{bg,x}}{TP_A+FP_A}\right)\right)\\
&\quad - \frac{w_{fg,y}}{w_{fg,y}+w_{bg,y}}\frac{w_{fg,y}\cdot FP_A - w_{bg,y}\cdot TP_A}{w_{fg,y}+w_{bg,y}}\left(\ln\left(1+\frac{w_{fg,y}+w_{bg,y}}{TP_A+w_{fg,x}+FP_A+w_{bg,x}}\right)-\ln\left(1+\frac{w_{fg,y}+w_{bg,y}}{TP_A+FP_A}\right)\right)\\
&\quad - \frac{w_{fg,y}}{w_{fg,y}+w_{bg,y}}\frac{w_{fg,y}\cdot w_{bg,x} - w_{bg,y}\cdot w_{fg,x}}{w_{fg,y}+w_{bg,y}}\cdot\ln\left(1+\frac{w_{fg,y}+w_{bg,y}}{TP_A+w_{fg,x}+FP_A+w_{bg,x}}\right)\\
&\quad + \frac{w_{fg,x}}{w_{fg,x}+w_{bg,x}}\frac{w_{fg,x}\cdot w_{bg,y} - w_{bg,x}\cdot w_{fg,y}}{w_{fg,x}+w_{bg,x}}\cdot\ln\left(1+\frac{w_{fg,x}+w_{bg,x}}{TP_A+w_{fg,y}+FP_A+w_{bg,y}}\right)\\
&= \frac{w_{fg,x}}{w_{fg,x}+w_{bg,x}}\frac{w_{fg,x}\cdot FP_A - w_{bg,x}\cdot TP_A}{w_{fg,x}+w_{bg,x}}\left(\ln\left(1+\frac{w_{fg,y}+w_{bg,y}}{TP_A+FP_A+w_{fg,x}+w_{bg,x}}\right)-\ln\left(1+\frac{w_{fg,y}+w_{bg,y}}{TP_A+FP_A}\right)\right)\\
&\quad - \frac{w_{fg,y}}{w_{fg,y}+w_{bg,y}}\frac{w_{fg,y}\cdot FP_A - w_{bg,y}\cdot TP_A}{w_{fg,y}+w_{bg,y}}\left(\ln\left(1+\frac{w_{fg,y}+w_{bg,y}}{TP_A+w_{fg,x}+FP_A+w_{bg,x}}\right)-\ln\left(1+\frac{w_{fg,y}+w_{bg,y}}{TP_A+FP_A}\right)\right)\\
&\quad + \left[\frac{w_{bg,y}}{w_{fg,y}+w_{bg,y}}\frac{w_{fg,x}}{w_{fg,x}+w_{bg,x}} - \frac{w_{bg,x}}{w_{fg,x}+w_{bg,x}}\frac{w_{fg,y}}{w_{fg,y}+w_{bg,y}}\right]\\
&\quad \left[\frac{w_{fg,y}(w_{fg,x}+w_{bg,x})}{w_{fg,y}+w_{bg,y}}\cdot\ln\left(1+\frac{w_{fg,y}+w_{bg,y}}{TP_A+w_{fg,x}+FP_A+w_{bg,x}}\right)+\frac{w_{fg,x}(w_{fg,y}+w_{bg,y})}{w_{fg,x}+w_{bg,x}}\cdot\ln\left(1+\frac{w_{fg,x}+w_{bg,x}}{TP_A+w_{fg,y}+FP_A+w_{bg,y}}\right)\right]\\
&= \left[FP_A\left(\left(\frac{w_{fg,y}}{w_{fg,y}+w_{bg,y}}\right)^2-\left(\frac{w_{fg,x}}{w_{fg,x}+w_{bg,x}}\right)^2\right)+TP_A\left(\frac{w_{fg,x}w_{bg,x}}{(w_{fg,x}+w_{bg,x})^2}-\frac{w_{fg,y}w_{bg,y}}{(w_{fg,y}+w_{bg,y})^2}\right)\right]\\
&\quad \cdot\left[\ln\left(1+\frac{w_{fg,y}+w_{bg,y}}{TP_A+FP_A}\right)-\ln\left(1+\frac{w_{fg,y}+w_{bg,y}}{TP_A+w_{fg,x}+FP_A+w_{bg,x}}\right)\right]\\
&\quad + \left[\frac{w_{bg,y}}{w_{fg,y}+w_{bg,y}}\frac{w_{fg,x}}{w_{fg,x}+w_{bg,x}} - \frac{w_{bg,x}}{w_{fg,x}+w_{bg,x}}\frac{w_{fg,y}}{w_{fg,y}+w_{bg,y}}\right]\\
&\quad \left[\frac{w_{fg,y}(w_{fg,x}+w_{bg,x})}{w_{fg,y}+w_{bg,y}}\cdot\ln\left(1+\frac{w_{fg,y}+w_{bg,y}}{TP_A+w_{fg,x}+FP_A+w_{bg,x}}\right)+\frac{w_{fg,x}(w_{fg,y}+w_{bg,y})}{w_{fg,x}+w_{bg,x}}\cdot\ln\left(1+\frac{w_{fg,x}+w_{bg,x}}{TP_A+w_{fg,y}+FP_A+w_{bg,y}}\right)\right]
\end{aligned}
$$

We substitute

$$
\begin{aligned}
c &= \left[\ln\left(1+\frac{w_{fg,y}+w_{bg,y}}{TP_A+FP_A}\right)-\ln\left(1+\frac{w_{fg,y}+w_{bg,y}}{TP_A+w_{fg,x}+FP_A+w_{bg,x}}\right)\right] > 0\\
p &= \frac{w_{fg,x}}{w_{fg,x}+w_{bg,x}}\\
q &= \frac{w_{fg,y}}{w_{fg,y}+w_{bg,y}}\\
w &= \frac{q(w_{fg,x}+w_{bg,x})\cdot\ln\left(1+\frac{w_{fg,y}+w_{bg,y}}{TP_A+w_{fg,x}+FP_A+w_{bg,x}}\right)+p(w_{fg,y}+w_{bg,y})\cdot\ln\left(1+\frac{w_{fg,x}+w_{bg,x}}{TP_A+w_{fg,y}+FP_A+w_{bg,y}}\right)}{c}
\end{aligned}
$$

and obtain

$$
\begin{aligned}
\Delta &= c\left(FP_A(q^2-p^2)+TP_A(p(1-p)-q(1-q))+(p-q)w\right)\\
&= c(p-q)\left(-FP_A(p+q)+TP_A(1-(p+q))+w\right)\\
&= c(p-q)\left(TP_A-(TP_A+FP_A)(p+q)+w\right) > 0
\end{aligned}
$$

leading to the conditions:

1. $p > q$ equivalent to $\frac{w_{fg,x}}{w_{fg,x}+w_{bg,x}} > \frac{w_{fg,y}}{w_{fg,y}+w_{bg,y}}$, which has been the premise of Lemma 3, and

2. $TP_A - (TP_A + FP_A)(p+q) + w > 0$, which is still to prove.

Hence, we finally prove $w > (TP_A + FP_A)(p+q)$.

$$w = \frac{q(w_{fg,x}+w_{bg,x}) \cdot \ln\left(1 + \frac{w_{fg,y}+w_{bg,y}}{TP_A+w_{fg,x}+FP_A+w_{bg,x}}\right) + p(w_{fg,y}+w_{bg,y}) \cdot \ln\left(1 + \frac{w_{fg,x}+w_{bg,x}}{TP_A+w_{fg,y}+FP_A+w_{bg,y}}\right)}{\ln\left(1 + \frac{w_{fg,y}+w_{bg,y}}{TP_A+FP_A}\right) - \ln\left(1 + \frac{w_{fg,y}+w_{bg,y}}{TP_A+w_{fg,x}+FP_A+w_{bg,x}}\right)}$$

We use

$$\ln\left(1 + \frac{e}{f+g}\right) > \frac{e}{e+f+g}$$
$$\ln\left(1 + \frac{e}{f}\right) - \ln\left(1 + \frac{e}{f+g}\right) < \frac{eg}{f(e+f+g)}$$

for $e, g > 0$ which is always fulfilled, since e ad g correspond to the sums of foreground and background weights, and data points without any weight assigned can be ignored.

$$\begin{aligned} w &> \frac{(q+p)\frac{(w_{fg,x}+w_{bg,x})(w_{fg,y}+w_{bg,y})}{TP_A+FP_A+w_{fg,x}+w_{bg,x}+w_{fg,y}+w_{bg,y}}}{\frac{(w_{fg,y}+w_{bg,y})(w_{fg,x}+w_{bg,x})}{(TP_A+FP_A)(TP_A+FP_A+w_{fg,x}+w_{bg,x}+w_{fg,y}+w_{bg,y})}} \\ &= (p+q)(TP_A+FP_A) \end{aligned}$$

Hence, there is only a single condition $p > q$, which is identical to $\frac{w_{fg,x}}{w_{fg,x}+w_{bg,x}} > \frac{w_{fg,y}}{w_{fg,y}+w_{bg,y}}$. □

Theorem 1. *Let D be a weighted data set of N data points and $(w_{fg,n}, w_{bg,n})$ be the weights for data point x_n. Furthermore, let c_n be the classification score of data point x_n assigned by a classifier, and let s be the order of classification scores, i.e.,*

$$\forall i \in [1, N-1] : c_{s_i} \leq c_{s_{i+1}}.$$

1. *The maximal AUC-PR is obtained iff the weights of the data points are monotonically increasing with respect to the sorting s, i.e.,*

$$\forall i \in [1, N-1] : \frac{w_{fg,s_i}}{w_{fg,s_i} + w_{bg,s_i}} \leq \frac{w_{fg,s_{i+1}}}{w_{fg,s_{i+1}} + w_{bg,s_{i+1}}}.$$

2. *The minimal AUC-PR is obtained iff the weights of the data points are monotonically decreasing with respect to the sorting s, i.e.,*

$$\forall i \in [1, N-1] : \frac{w_{fg,s_i}}{w_{fg,s_i} + w_{bg,s_i}} \geq \frac{w_{fg,s_{i+1}}}{w_{fg,s_{i+1}} + w_{bg,s_{i+1}}}.$$

Proof. Here, we only prove maximal AUC-PR, the minimal AUC-PR can be proven identically. The proof goes by contradiction.

Assume there is another sorting D of the data points that yields the maximal AUC-PR α_D. Since the data points are not ordered increasingly according to $\frac{w_{fg,n}}{w_{fg,n}+w_{bg,n}}$, there is at least one position i with $\frac{w_{fg,i}}{w_{fg,i}+w_{bg,i}} < \frac{w_{fg,i+1}}{w_{fg,i+1}+w_{bg,i+1}}$. Based on Lemma 3, we can exchange the order of the data points i and $i+1$ with their corresponding weights and will obtain a new sorting E with AUC-PR $\alpha_E > \alpha_D$. Hence, the initial ordering D does not yield the maximal AUC-PR. □
